# The Hyperdense Right Hemidiaphragm Sign: A Novel Radiological Indicator of Diffuse Fatty Liver Infiltration on Non-Enhanced CT

**DOI:** 10.7759/cureus.75340

**Published:** 2024-12-08

**Authors:** Anwaar Al Maawali, Ishaq Al Salmi, Tahra Al Saadi, Hussain Al Balushi

**Affiliations:** 1 Radiology, Rustaq Hospital, Rustaq, OMN; 2 Radiology, Royal Hospital, Muscat, OMN; 3 Radiology, Armed Forces Hospital, Muscat, OMN

**Keywords:** fatty infiltration, hepatic steatosis, hyperdense right hemidiaphragm, liver, mri

## Abstract

Objectives

The primary objective of this study is to describe and evaluate the diagnostic performance of the hyperdense right hemidiaphragm sign (HRHS) as a novel radiological indicator for diffuse fatty infiltration of the liver on non-enhanced CT (NECT) scans. This includes assessing its sensitivity, specificity, positive predictive value, and negative predictive value, and comparing these metrics with other established NECT signs.

Methods

This cross-sectional multicenter retrospective study included all patients over 12 years of age who underwent both abdominal MRI and NECT scans of the abdomen within a period not exceeding six months at two tertiary hospitals (The Royal Hospital and Armed Forces Hospital, Muscat, Sultanate of Oman) between January 2010 and December 2022. Two readers reviewed the NECT scan images for the following signs: HRHS, liver density of less than 10 HU compared to the spleen, absolute liver density of <40 HU, and hyperattenuated intrahepatic vessels. The drop in signal on the out-phase sequence compared to the in-phase sequence on MRI was used as the gold standard reference for diagnosing diffuse fatty infiltration of the liver. Data were analyzed using IBM SPSS Statistics for Windows, Version 26.0 (Released 2019; IBM Corp., Armonk, NY, USA) and MedCalc software (MedCalc Software Ltd., Oostende, Belgium), with sensitivity, specificity, and positive and negative predictive values calculated for each NECT sign.

Results

A total of 340 patients were included in this study, with a mean age of 54 years. Among these patients, 45 were diagnosed with diffuse fatty infiltration of the liver by MRI. Thirty-six patients were identified as positive for fatty liver infiltration on unenhanced CT based on the criterion of the HRHS, with a sensitivity of 91%, specificity of 99%, positive predictive value of 94%, and negative predictive value of 99%. In contrast, 45 patients were positive using the criterion of liver density less than 10 HU compared to the spleen, yielding a sensitivity of 83%, a specificity of 98%, a positive predictive value of 88%, and a negative predictive value of 97%. Using the criterion of absolute liver density <40 HU, 30 patients were positive, with a sensitivity of 72%, specificity of 98%, positive predictive value of 90%, and negative predictive value of 96%. Lastly, 20 patients were positive using the criterion of hyperattenuated intrahepatic vessels, with a sensitivity of 51%, specificity of 100%, positive predictive value of 100%, and negative predictive value of 93%.

Conclusions

The proposed qualitative evaluation using the HRHS for diffuse fatty infiltration of the liver on NECT scans demonstrates the highest sensitivity compared to other previously described NECT signs.

## Introduction

Hepatic steatosis is a progressive condition with a rising global prevalence, and it is associated with various hepatic and extrahepatic complications [1]. Several risk factors contribute to the development of fatty liver, including obesity, alcohol consumption, parenteral nutrition, and certain chemotherapeutic medications. It is a common condition that typically presents without symptoms [2]. The condition is caused by the accumulation of lipid metabolites in the liver, including triglycerides in most cases, as well as free fatty acids and cholesterol. When no specific cause is identified, the condition is referred to as non-alcoholic fatty liver disease (NAFLD). Potential causes of hepatic steatosis include alcohol abuse, steatogenic medications, and viral infections. NAFLD is considered the most prevalent chronic liver disease, affecting approximately 25% of the global population. The highest prevalence rates are observed in the Middle East (32%), South and North America (30% and 24%, respectively), and Asia (27%), as reported by Starekova et al. [1].

Isolated hepatic steatosis is known to have significant clinical implications. Numerous studies have shown a strong association between metabolic syndrome, cardiovascular diseases, and hepatic steatosis. It is also linked to the development of type 2 diabetes. Among patients with NAFLD, the prevalence of diabetes and metabolic syndrome is estimated to be 23% and 41%, respectively. NAFLD is also associated with a higher prevalence of coronary artery disease and atherosclerosis (ORs: 1.9 and 1.3, respectively), as noted in the study by Starekova et al. [1].

Hepatic steatosis is known to influence the progression of hepatic fibrosis and the response to antiviral treatments in patients with hepatitis C. Moreover, it plays a crucial role as a cofactor in liver injury observed in patients with hemochromatosis and alcoholic liver disease [3,4]. Given the rising obesity epidemic and the advent of new therapeutics aimed at altering metabolism, there is an increasing need to quantify and monitor liver steatosis [5].

While histologic examination remains the most accurate method for detecting hepatic fat content, it is invasive, time-consuming, and prone to sampling errors. The most reliable method for detecting and grading steatosis is liver biopsy, although it is non-targeted. The earliest histological feature of NAFLD is the intracellular accumulation of triglycerides within hepatocytes, which is considered a hallmark of the disease. This fat accumulation leads to oxidative stress, which can contribute to liver injury, inflammation, and fibrosis [6]. Grading of hepatic steatosis is determined by the percentage of hepatocytes containing lipid-filled vacuoles. According to Starekova et al. and Brunt et al., grade 0 (66% of hepatocytes affected) is classified as severe. A threshold of 30% is typically used to indicate moderate steatosis. It is important to note that these histopathologic thresholds are based on visual assessments, with no clear correlation to prognosis or other clinical outcomes [1,2].

Several imaging methods can detect hepatic steatosis, ranging from simple qualitative techniques to more complex but highly accurate methods [6]. Sonography, MRI, and CT are non-invasive modalities used for fat detection. Sonography is the simplest method but is limited to qualitative assessments. MRI is the most accurate for detecting even small amounts of fatty infiltration, although it is relatively expensive. CT is widely accepted as the standard imaging modality for steatosis detection [3].

In CT imaging, fatty infiltration is identified by a decrease in CT attenuation, as triglycerides have lower X-ray absorption than normal liver tissue. The degree of fatty infiltration correlates with the reduction in CT attenuation values. These values can be measured in several ways, including hepatic attenuation alone, or by normalizing hepatic attenuation with splenic attenuation, calculating the ratio between these values [1,2].

For unenhanced CT, several diagnostic methods have been proposed over the years. Some researchers suggest comparing liver and spleen CT values as an internal standard, as other materials like iron can affect X-ray attenuation. Additionally, variations in CT scanner calibration and manufacturer-specific differences in Hounsfield units (HU) may influence results [4]. The sensitivity and specificity of CT for mild steatosis (10-20% based on liver biopsy) are 57% and 88%, respectively, while for higher-grade steatosis (>25%), sensitivity rises to 72%, and specificity increases to 95%. A threshold of 48 HU on unenhanced CT has been shown to be highly specific (100%) for moderate-to-severe steatosis (≥30%) with a sensitivity of 54%, a positive predictive value of 100%, and a negative predictive value of 94%, as reported by Starekova et al. [1].

No single method has proven superior for diagnosing steatosis on unenhanced CT. One approach involves measuring the absolute liver attenuation with a threshold ≤40 HU, which demonstrates high specificity, although with low sensitivity. Another method calculates the liver-to-spleen attenuation ratio, but this may increase the risk of false positives. Fat sparing on CT, identified by a characteristic pattern, is an unequivocal sign of steatosis, indicating the presence of fatty infiltration in at least some parts of the liver [5].

Despite its diagnostic capabilities, CT is not typically used for the initial assessment of liver steatosis due to its associated risk of ionizing radiation and its lower sensitivity for detecting mild steatosis. MRI provides a safer and more reliable alternative [4].

MRI can detect and quantify fat in the liver by measuring proton signals from water and fat. In-phase and opposed-phase imaging or fat-suppression techniques (such as T1-weighted gradient-echo and T2-weighted fast spin-echo sequences) are widely used for qualitative assessment of hepatic steatosis [1]. On MRI, fatty liver appears with high signal intensity on T1-weighted images, making it easily detectable by visual inspection [4]. Among MRI techniques, magnetic resonance spectroscopy is considered the most accurate method for fat detection and quantification, although it is expensive, and the necessary software is not universally available. MR elastography, which is a newer method for detecting liver stiffness, is still under investigation for its ability to detect fat [4].

MRI has some drawbacks, such as sensitivity to motion and parallel imaging artifacts, which can affect the accuracy of measurements, particularly when selecting regions of interest [3,6-8].

Early diagnosis of liver fat infiltration is critical for effective management and better patient outcomes. However, many of the proposed non-enhanced CT (NECT) radiological signs used to detect hepatic fat infiltration require the calculation of HU [5,9-12].

In our clinical practice, we observed that patients with diffuse fatty infiltration of the liver, and consequently low attenuation of the liver parenchyma on NECT scans, showed a slight hyperdensity of the right hemidiaphragm. In normal subjects, the liver parenchyma and the right hemidiaphragm exhibit nearly identical attenuation on NECT, making them indistinguishable. However, in patients with diffuse hepatic fat deposition, the liver attenuation is reduced, resulting in a noticeable contrast between the liver parenchyma and the adjacent right hemidiaphragm. In these patients, the right hemidiaphragm appears brighter than the liver parenchyma. Based on this observation, we propose a new radiological sign: the hyperdense right hemidiaphragm sign (HRHS). In this study, we aim to assess the utility of HRHS and compare its diagnostic performance with preexisting radiological signs.

To the best of our knowledge, no previous studies have described or evaluated the diagnostic performance of this sign. The advantage of HRHS lies in its speed and ease of detection by visual inspection, which contrasts with other methods that require HU measurements of the liver and spleen.

The primary objective of this study is to introduce HRHS as a novel radiological sign for diagnosing diffuse hepatic fat infiltration on NECT scans and to evaluate its sensitivity, specificity, positive predictive value, and negative predictive value in comparison with established NECT signs.

## Materials and methods

This cross-sectional multicenter retrospective study included all patients above 12 years of age who underwent both abdominal MRI and NECT scans of the abdomen within a six-month period. The study was not randomized, and all subjects who met the inclusion and exclusion criteria were included. It was conducted across two tertiary hospitals, The Royal Hospital and Armed Forces Hospital, in Muscat, Sultanate of Oman, between January 2010 and December 2022. Data was collected from both institutions, with a total of 340 patients included in the study.

**Figure 1:**
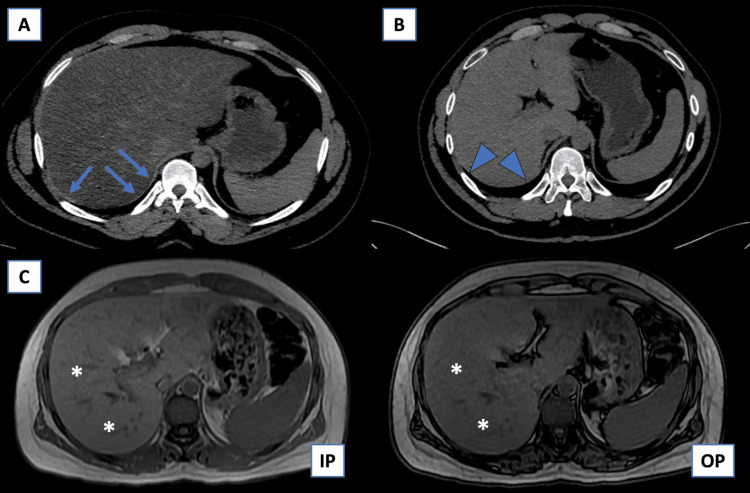
Example of the HRHS in a patient with diffuse fatty liver infiltration (a) NECT scan of a patient with diffuse fatty infiltration of the liver, demonstrating a positive HRHS (arrow), where the diaphragm appears hyperattenuating compared to the adjacent liver parenchyma. (b) NECT scan of a patient with normal liver parenchyma and a negative HRHS, where both the liver and the right hemidiaphragm exhibit similar attenuation and cannot be distinguished (arrowhead). (c) T1 IP (on the left) and OP (on the right) sequences of the same patient in (a), showing signal dropout in the out-phase sequence (asterisk), consistent with diffuse fatty infiltration of the liver. HRHS, hyperdense right hemidiaphragm sign; IP, in-phase; NECT, non-enhanced CT; OP, out-phase

Demographic data, including age and gender, were retrieved from the Al-Shifa Hospital Information System after obtaining ethical approval from the ethical committees of both hospitals. Radiological data were collected from the Picture Archiving and Communication System (PACS) and analyzed using IBM SPSS Statistics for Windows, Version 26.0 (Released 2019; IBM Corp., Armonk, NY, USA) and MedCalc software (MedCalc Software Ltd., Oostende, Belgium). NECT scan images were reviewed and analyzed by two readers from each institution, focusing on the following signs: the HRHS (Figure 1), liver density less than 10 HU compared to the spleen, absolute liver density <40 HU, and hyperattenuated intrahepatic vessels.

MRI was considered the gold standard for confirming fatty infiltration of the liver [2,3]. All MRI examinations were reviewed by the assigned radiologists, who evaluated them carefully for evidence of fatty infiltration while being blinded to the CT scan results. A drop in signal on the out-phase sequence compared to the in-phase sequence in MRI is considered diagnostic for fatty infiltration. The signal intensity drop was quantified by measuring the actual signal intensity difference between the out-phase and in-phase images in both the liver and spleen. The percentage of signal intensity loss (PSIL) was calculated using the following formula: [(Liver IP / Spleen IP) - (Liver OOP / Spleen OOP)] / [(Liver IP / Spleen IP)] x 100. A PSIL of more than 10% was considered indicative of fatty liver infiltration [4,5].

For patients who underwent both plain and post-contrast CT scans of the abdomen in the portal venous phase, the HRHS was also assessed on contrast-enhanced CT (CECT) images. Sensitivity, specificity, as well as positive and negative predictive values, were then calculated.

## Results

During the study period, a total of 340 patients were included, of which 182 (53.5%) were male and 158 (46.5%) were female. The average age of male patients was 54.9 years, while for female patients, it was 55.0 years.

Out of the 340 patients, 45 were diagnosed with diffuse fatty infiltration of the liver by MRI. Among these, 36 patients demonstrated a positive HRHS for fatty liver infiltration on NECT, with a sensitivity of 91%, specificity of 99%, positive predictive value of 94%, and negative predictive value of 99%. Using the preexisting NECT sign of liver density less than 10 HU compared to the spleen, 45 out of 340 patients were positive for fatty liver infiltration, with a sensitivity of 83%, specificity of 98%, positive predictive value of 88%, and negative predictive value of 97%. A criterion of liver density <40 HU identified 30 positive patients, with a sensitivity of 72%, specificity of 98%, positive predictive value of 90%, and negative predictive value of 96%. Only 20 patients were positive for fatty liver infiltration using the criterion of hyperattenuated intrahepatic vessels, with sensitivity, specificity, positive predictive value, and negative predictive value of 51%, 100%, 100%, and 93%, respectively. Additionally, only three patients were positive for fatty liver infiltration using the HRHS criterion in CECT, with a sensitivity of 11%, specificity of 100%, positive predictive value of 100%, and negative predictive value of 91%. These results are summarized in Table 1. There was 100% concordance between the two readers for the proposed HRHS.

**Table 1:** Summary of the sensitivity, specificity, positive predictive value, and negative predictive value of the HRHS and other established NECT signs for diffuse fatty liver infiltration The last column includes these parameters for the HRHS on CECT. CECT, contrast-enhanced CT; HRHS, hyperdense right hemidiaphragm sign; NECT, non-enhanced CT

Signs used for hepatic fat detection on CT	HRHS on unenhanced CT	Liver density less than 10 HU compared to spleen	Liver density <40 HU	Hyperattenuated intrahepatic vessels	HRHS in CECT
Sensitivity	91%	83%	72%	51%	11%
Specificity	99%	98%	98%	100%	100%
Positive predictive value	94%	88%	90%	100%	100%
Negative predictive value	99%	97%	96%	93%	91%

## Discussion

Liver cirrhosis is a serious disorder with a high risk of complications, including varices, ascites, hepatic encephalopathy, hepatopulmonary hypertension, hepatocellular carcinoma, hepatorenal syndrome, spontaneous bacterial peritonitis, and coagulation disorders. As a result, liver cirrhosis ranks as the 11th leading cause of death worldwide, with an estimated two million deaths per year attributed to the condition. Projections suggest that these numbers will increase in the future [9].

Diffuse fatty infiltration of the liver is an early indicator of hepatocyte injury, which can be reversible if detected and managed promptly [10]. In contrast, other radiological features of cirrhosis, except for elastography, typically reflect late-stage damage, often making the condition irreversible. Therefore, detecting early signs of cirrhosis is crucial in preventing progression to advanced stages and minimizing complications [11,12].

Radiologists play a key role in identifying early features of chronic liver disease, which may present as diffuse fatty liver infiltration. This finding is often incidental in patients being evaluated for other abdominal pathologies. CT scans are commonly employed to assess various abdominal conditions, with NECT being used either as a dedicated examination or in combination with contrast-enhanced imaging, depending on the clinical situation. Additionally, NECT can be virtually derived from post-contrast scans if dual-energy CT technology is used.

While many radiological signs for assessing fatty liver infiltration on NECT have been described in the literature, most require time-consuming measurements of the average HU of the liver and spleen. This is not feasible in patients who have undergone splenectomy or have widespread splenic disease. As a simpler alternative, we propose a new radiological sign on NECT, the HRHS, which was observed in our clinical practice. This study aims to evaluate the diagnostic performance of this sign in comparison with other established signs. The HRHS is based on the fact that both liver parenchyma and skeletal muscles (including the diaphragm) typically have similar attenuation on NECT, making them difficult to differentiate. However, in cases of diffuse fatty liver infiltration, the liver parenchyma becomes hypoattenuated and appears darker, while the right hemidiaphragm appears brighter, or hyperdense, relative to the liver.

Our study shows promising results for the HRHS on NECT, with high sensitivity (91%), specificity (99%), positive predictive value (94%), and negative predictive value (99%) for diagnosing diffuse fatty liver infiltration. These results surpass those of other established signs. Notably, all NECT signs demonstrate high specificity (98% or greater) for detecting fatty liver infiltration. While HRHS on CECT at the portal venous phase is highly specific (100%) for diagnosing diffuse fatty liver infiltration, it is not applicable for other signs.

The higher sensitivity of HRHS compared to other NECT signs suggests that it can detect even mild cases of diffuse fatty liver infiltration without sacrificing specificity. This feature may be particularly useful for patients who have undergone splenectomy or those with diffuse splenic disease. The lower sensitivity of HRHS, when compared to MRI chemical shift imaging (the gold standard modality for fatty liver detection), may be due to MRI’s ability to detect very mild fatty infiltration, where the attenuation difference between the liver and diaphragm is less pronounced. The small thickness of the diaphragm, required to appreciate its attenuation difference relative to the liver, may also contribute to this limitation.

Our findings for other NECT signs are consistent with the literature. The sensitivity of these signs in our study ranged from 51% to 83%, which aligns with the range of 43% to 95% found in a study by Dendl and Schreyer. However, our study shows higher specificity (98-100%) compared to the 90% specificity reported in Dendl and Schreyer’s study [7].

The main limitation of our study is the lack of histopathological confirmation of diffuse fatty liver infiltration, which was not feasible. Additionally, the relatively small number of patients diagnosed with diffuse fatty infiltration in our sample may influence the results.

The HRHS demonstrated high sensitivity and specificity for diagnosing diffuse fatty liver infiltration, and larger-scale studies are needed to confirm the reproducibility of this sign. Further research could also explore the use of HU measurements between the liver and skeletal muscles to enhance sensitivity. It is important to note that HRHS cannot be used to diagnose focal fatty infiltration of the liver, which is a limitation shared by other CT signs for diffuse fatty infiltration.

## Conclusions

The HRHS demonstrates both high specificity and sensitivity in diagnosing diffuse fatty liver infiltration on NECT. Our results indicate that HRHS outperforms other previously described NECT signs in terms of sensitivity, making it a valuable diagnostic tool for identifying fatty liver infiltration.
